# Hypertrophic Cardiomyopathy in a Latin American Center: A Single Center Observational Study

**DOI:** 10.3390/jcm12175682

**Published:** 2023-08-31

**Authors:** Juan David López-Ponce de Leon, Mayra Estacio, Natalia Giraldo, Manuela Escalante, Yorlany Rodas, Jessica Largo, Juliana Lores, María Camila Victoria, Diana Argote, Noel Florez, Diana Carrillo, Pastor Olaya, Mauricio Mejia, Juan Esteban Gomez

**Affiliations:** 1 Departamento de Cardiología, Fundación Valle del Lili, Cali 760032, Colombia; natalia.giraldo@fvl.org.co (N.G.); noel.florez@fvl.org.co (N.F.); diana.carrillo@fvl.org.co (D.C.); pastor.olaya@fvl.org.co (P.O.); juan.gomez@fvl.org.co (J.E.G.); 2 Facultad de Ciencias de la Salud, Universidad Icesi, Cali 760031, Colombiadiana.argote.ri@fvl.org.co (D.A.); 3 Centro de Investigaciones Clínicas, Fundación Valle del Lili, Cali 760032, Colombia; mayraestacio1@gmail.com (M.E.); manuela.escalante@fvl.org.co (M.E.); jessica.largo.oc@fvl.org.co (J.L.); camilavictoriareyes@gmail.com (M.C.V.); 4 Departamento de Radiología, Fundación Valle del Lili, Cali 760032, Colombia; mauricio.mejia@fvl.org.co

**Keywords:** cardiomyopathies, hypertrophic cardiomyopathy

## Abstract

Background: Hypertrophic cardiomyopathy (HCM) is a complex disorder that includes various phenotypes, leading to different manifestations. It also shares different disadvantages typical of rare diseases, including limited recognition, lack of prospective studies assessing treatment, and little or delayed access to advanced treatment options. Reliable data about the prevalence and natural history of cardiomyopathies in South America are lacking. This study summarizes the features and management of patients with HCM in a university hospital in Colombia. Methods: This was an observational retrospective cohort study of patients with HCM between January 2010 and December 2021. Patient data were analyzed from an institutional cardiomyopathy registry. Demographic, paraclinical, and outcome data were collected. Results: A total of 82 patients during the study period were enrolled. Of these, 67.1% were male, and the mean age at diagnosis was 49 years. Approximately 83% were in NYHA functional class I and II, and the most reported symptoms were dyspnea (38%), angina (20%), syncope (15%), and palpitations (11%). In addition, 89% had preserved left ventricular ejection fraction (LVEF) with an asymmetric septal pattern in 65%. Five patients (6%) had alcohol septal ablation and four (5%) had septal myectomy. One patient required heart transplantation during follow-up. Sudden cardiovascular death was observed in 2.6%. The overall mortality during follow-up was 7.3%. Conclusions: HCM is a complex and heterogeneous disorder that presents with significant morbidity and mortality. Our registry provides comprehensive data on disease courses and management in a developing country.

## 1. Introduction

Cardiomyopathies are a heterogeneous group of disorders in which the heart muscle is structurally and functionally abnormal in the absence of obstructive coronary artery disease, hypertension, valvular disease, and congenital heart disease sufficient to explain the observed myocardial abnormality [1,2]. The main phenotypes are dilated cardiomyopathy (DCM), hypertrophic cardiomyopathy (HCM), restrictive cardiomyopathy (RCM), left ventricular noncompaction (LVNC), and arrhythmogenic cardiomyopathy (ACM) [3]. In general, cardiomyopathy is an important problem, as it is associated with sudden death in young adults and is one of the main causes of heart transplantation.

The incidence and prevalence of inherited cardiomyopathies have been derived from screening studies and can vary by type and by geographic region. Reliable epidemiology of cardiomyopathies is primarily accessible from developed nations where accurate prevalence statistics based on the use of established diagnostic evaluations and criteria are collected [4,5].

HCM is a genetic disorder of cardiac myocytes characterized by unexplained cardiac hypertrophy without the presence of other pathologies that increase loading conditions. For a clinical diagnosis, a left ventricle (LV) wall thickness in diastole >15 mm must be present or >13 mm if there is a family member with HCM. A disease prevalence of 1:250 to 500 for HCM in adults seems to be similar in all races, and disease expression usually occurs in adolescents and young adults [4]. Unfortunately, South America lacks reliable data about the prevalence and incidence of cardiomyopathies [6,7].

In relation to the information on hypertrophic cardiomyopathy in Latin America, there are two studies, the first carried out in Argentina by Fernandez et al. in which they evaluated prevalence, clinical course, and pathological findings of left ventricular systolic impairment in patients with HCM [8] and the second in which Nilda Espínola-Zavaleta et al. in Mexico evaluated the survival and clinical behavior of hypertrophic cardiomyopathy in a cohort [9].

Cardiomyopathies are associated with high morbidity and mortality associated with premature death from arrhythmia, progressive heart failure, or stroke [10]. Most information about the presentation and natural history of cardiomyopathies has been derived from cohort studies in Europe and North America [11,12,13]. Information about the clinical profile and management of the disease at a national level is very limited.

This study summarizes the features and management of patients with HCM in a center that provides highly complex health services in Colombia.

## 2. Methods

This is an observational retrospective cohort study of patients with HCM that are included in the institutional cardiomyopathy registry (RIM) that have information on five cardiomyopathy subtypes, DCM, HCM, LVNC, ARVC, and RCM, diagnosticated between January 2010 and December 2021 in Fundación Valle del Lili in Cali, Colombia.

The patients with HCM had to meet the following criteria: evidence of left ventricular hypertrophy with a wall thickness of ≥15 mm (or >13 mm if there is a family member with HCM) in one or more myocardial segments in the absence of loading conditions, such as hypertension or valve disease, documented by echocardiography or cardiac magnetic resonance imaging (CMR) [14]. Eighty-two patients (27%) of the registry met the inclusion criteria for HCM.

Information obtained from the database included demographics, clinical and paraclinical comorbidities (NT-proBNP, Troponin, electrocardiogram), family history for HCM or SCD, and pharmacological and non-pharmacological treatments. The comorbidities evaluated were atrial fibrillation, stroke, diabetes mellitus, arterial hypertension, dyslipidemia, overweight, obesity, chronic kidney disease, hypothyroidism, and smoking. NYHA functional class and symptoms, such as angina, dyspnea, palpitations, and syncope, were recorded from the clinical history of admission.

Positive family history for HCM was defined as the documented presence of the disease in a first-degree relative, whereas positive family history for sudden cardiac death (SCD) was defined as the unexpected death of a first-degree relative younger than 40 years old. Cardiac dimensions and function were based on echocardiographic and CMR measurements.

All included patients had at least one follow-up visit, and those visits varied in timeframes for each patient. Atrial fibrillation and ventricular tachycardia (sustained or not sustained) were diagnosed based on an electrocardiogram (ECG) or 24 h ECG monitor recording or by an established history of the arrhythmias. Interpretations of the 12-lead electrocardiogram and 24 h ECG monitor were performed by a cardiologist or electrophysiologist. The outcomes of the study were mortality by any cause during the follow-up period, including SCD.

Institutional echocardiograms were performed following the ASE guidelines [15]. Extra institutional echocardiograms could not be evaluated in relation to the performance techniques used.

The baseline echocardiogram includes a screening assessment of ventricular function, chamber sizes, left ventricular wall thicknesses, aortic root diameter, pericardial effusions, and gross valvular structure and function, including an estimate of pulmonary arterial systolic pressure using the peak tricuspid regurgitation velocity.

**Figure 1:**
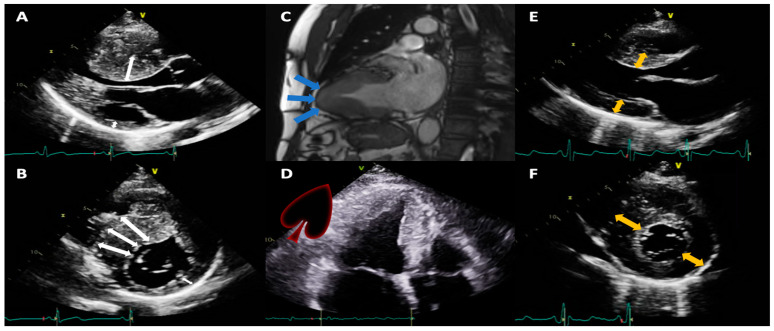
Echocardiographic and CMR images representing various phenotypic expressions of hypertrophic cardiomyopathy (**A**,**B**) from a patient with septal asymmetric hypertrophy (white double-headed arrows); (**C**) (CMR) and (**D**) in a patient with an apical HCM, which in systole produces a classic spade-shaped ventricular cavity (blue arrows); (**E**,**F**) in a concentric phenotype (yellow double-headed arrows). CMR: magnetic resonance imaging; HCM: hypertrophic cardiomyopathy.

The highest end-diastolic wall thickness was measured in the parasternal long-axis view and correlated with the same segment in the parasternal short-axis view, depending on the MHC phenotype, to avoid overestimations in quantification. They were classified depending on the location of the highest thickening and the number of segments involved in asymmetric septal, concentric, and predominantly apical. A description of the echocardiographic findings was made regarding the presence of primary mitral regurgitation, number, and location of papillary muscles, presence of anterior systolic movement of the SAM mitral valve, dynamic obstruction of the left ventricular outflow tract, or the presence of significant midventricular gradient. Echocardiographic phenotypes can be seen in Figure 1. Apical aneurysms and left ventricular thrombus were diagnosed by echocardiography or CMR.

Most of the CMRs were performed in the institution, using either 1.5 or 3.0 Tesla CMR scanners. LV measures of geometry and function were analyzed using standardized protocols. The asymmetric LV wall thickness was measured using the maximal end-diastolic wall thickness divided by the indexed LV end-diastolic volume (wall thickness/volume ratio), a useful discriminator between wall thickening due to exercise, pathological thickening-related HCM, or increased afterload conditions [14,15]. Myocardial T1 mapping was used to assess for diffuse myocardial fibrosis.

The patient data and laboratory findings were collected from the institutional medical records; each patient had an assigned ID number for confidentiality, and information was stored in REDCap (Research Electronic Data Capture). The study protocol was approved by the Institutional Review Board Ethics Committee of the Fundación Valle del Lili.

### Statistical Analyses

A univariate descriptive analysis was performed. The normality of the continuous variables was analyzed using the Shapiro–Wilk test, with a statistical significance level of 5%. Variables that did not meet the normality assumption are presented as median and interquartile range (RIC) and the remaining variables as mean and standard deviation. Qualitative variables are presented as absolute frequencies and percentages. Data analysis was performed with the statistical software R V.4.2.1 (R Foundation for Statistical Computing) through RStudio 2022.07.0+548.

## 3. Results

**Figure 2:**
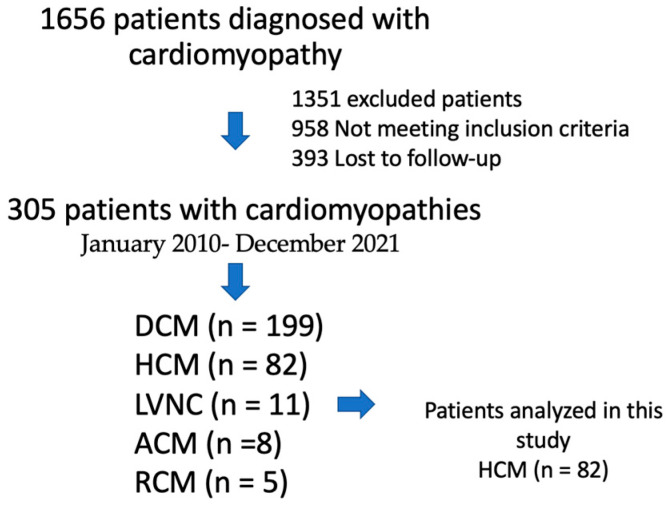
Flowchart describing patients in the study.

**Figure 3:**
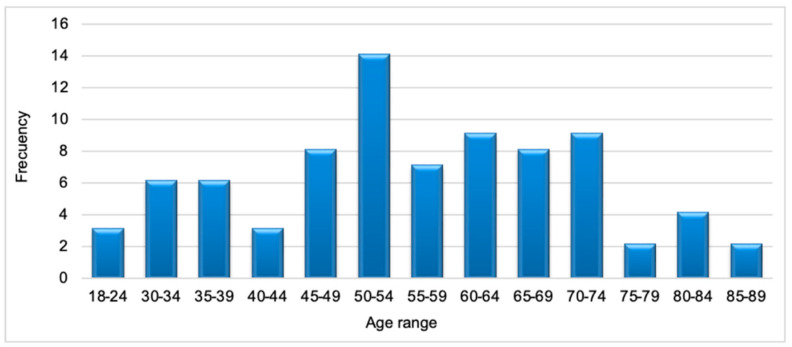
Age at the time of diagnosis.

Three hundred-five patients with cardiomyopathies were enrolled in the registry. Most patients had DCM (*n* = 199), followed by HCM (*n* = 82), LVNC (*n* = 11), ACM (*n* = 8), and RCM (*n* = 5) (Figure 2). In this study, 82 patients with HCM met the inclusion criteria. Baseline characteristics and outcomes are summarized in Table 1. The median age at diagnosis was 49 years (IQR 38–61), and most patients were male (67%) Figure 3.

The most frequent comorbidities were hypertension (30%), dyslipidemia (21%), atrial fibrillation (15%), type 2 diabetes mellitus (11%), stroke (9.5%), overweight or obesity (8%), hypothyroidism (8%), and chronic kidney disease (4%). Approximately 25% of the patients reported a family history of cardiomyopathy of which 67% were first-degree relatives, and 26% reported a history of sudden cardiac death (SCD) in any family member.

At enrollment, 60 patients (83%) were in the New York Heart Association (NYHA) I-II functional class. The most common symptoms were dyspnea (38%), angina (20%), syncope (15%), and palpitations (11%). Approximately 58% of the patients had an electrocardiogram record; the most frequent finding was atrial fibrillation (AF) in 12.5% and ventricular hypertrophy in only 8%. The NT-proBNP was measured in only 11 patients (13%), with a value > 1.200 pg/mL in 54.5% of the cases (negative if 125 pg/mL).

Table 1 describes pharmacologic and nonpharmacologic treatment before enrollment. Beta-blockers were the most frequently prescribed treatment (85%), followed by angiotensin-converting enzyme inhibitors (ACEi) and angiotensin II receptor blockers (ARBs) (26.5%). Sodium-glucose cotransporter-2 (SGLT-2) inhibitors were used in 1.5% of patients and angiotensin receptor-neprilysin inhibitors (ARNI) in 2.9%. For non-pharmacological therapy, the use of an implantable cardioverter-defibrillator was reported in 29 patients (35%); five patients (6%) underwent alcohol septal ablation; and four patients (5%) underwent septal myectomy.

The echocardiographic characteristics are described in Table 2. Most patients had a preserved ejection fraction (89%). Atrial dilatation > 34 mL/m^2^ was documented in 45 patients (85%), and resting or provoked left ventricular outflow obstruction (LVOTO) with a gradient > 30 mmHg was present in 29 (52.7%) patients. Systolic anterior motion (SAM) of the mitral valve was present in 21 (47.7%) patients. The most common pattern was asymmetric septal hypertrophy in 37 (64.9%) patients, followed by concentric hypertrophy in 16 (28.1%) and only four (7%) patients with a predominantly apical pattern. One patient had a left ventricular thrombus, and none had an apical aneurysm formation.

CMR was performed on 34% of the patients. The predominant pattern was also asymmetric septal hypertrophy in 64%, concentric hypertrophy in 7.4%, and apical hypertrophy in 14.8%. SAM was present in five (22.7%) patients. Wall thickness/volume ratio > 0.15 mm × m^2^ × mL (−1) was present in all patients.

Targeted sequencing of HCM genes and defining pathogenic variants was performed only in three patients who were sequenced using Illumina MiSeq (v2 kit) or NextSeq 500 (Mid Output v2 kit). The variants of genes encoded were TTN, TNNI3, and TTR.

During follow-up, heart transplantation was performed in one patient. SCD was observed in 2.6%. A history of AF was recorded in 22% of patients, and eight (10.7%) patients had an episode of sustained ventricular tachycardia. Eight patients were hospitalized during this period due to heart failure. The overall mortality during follow-up was 7.3% (six patients).

## 4. Discussion

This study provides a detailed contemporary assessment of the clinical profile, management strategies, and outcomes of HCM in a Latin American center. The clinical spectrum of HCM is complex and includes a variety of phenotypes, leading to different manifestations.

The most common type of LV hypertrophy was asymmetrical (64.9%), followed by concentric (28.1%) and apical (7.0%), with a similar trend to other studies [16]. Apical hypertrophic cardiomyopathy (AHCM) is a rare form of HCM that usually involves the left ventricle’s apex and is also known as Yamaguchi syndrome. Asian countries exhibit the highest prevalence of AHCM. Historically, this condition was thought to be confined to this population, but it is also found in other populations [17]. The prevalence of AHCM in China is reported to be as high as 41% and more than 15% in Japan, whereas in the USA, the prevalence is 1–3% [18]. In our study, AHCM was relatively common (7%), in Latin America; there are few studies evaluating the frequency of the disease as well as the predominant phenotypic expression. In 2015, Nilda Espinola-Zavaleta et al. reported a cohort of 77 Mexican patients with HCM, finding that 11% had an apical phenotype and this was associated with poorer survival [9]. In this sense, more studies are needed to determine the outcomes in this subgroup since in series from other regions of the world, such as Asian populations, apical HCM is an atypical phenotype and usually has an apparently benign course.

LVOTO is described in the literature with a prevalence near 70%, addressing the importance of provocation maneuvers that augment the gradient to >30 mmHg in more than half of the patients with low gradients at rest [19,20]. Our LVOTO had a prevalence of only 52.7%, but considering the type of study, we cannot assure all gradients were evaluated in resting and provoked conditions with either Valsalva maneuvers or stress echocardiography, also taking into consideration that this variable is highly dynamic and influenced by factors that alter cardiac contractility and loading conditions.

Most patients received medical treatment. The proportion of patients on diuretics and ARBs was high although most of the patients had preserved LVEF; 30% had arterial hypertension. Current U.S. and European guidelines recommend ACEi and ARBs as a suitable first choice for hypertension treatment together with calcium channel blockers and thiazide diuretics [21]. Alcohol septal reduction (ASA) therapy was performed in 8% of our cohort, and 4.9% had a surgical septal myectomy. In adult patients with obstructive HCM who remain severely symptomatic despite medical treatment, septal reduction therapies are indicated. Even though recent studies keep showing better outcomes with septal myectomy in eligible patients in whom surgery is contraindicated or the risk is considered unacceptable because of comorbidities or advanced age, ASA is an option in experienced centers [22]. Despite these recommendations, 43% of United States patients undergo ASA instead of myectomy, and these numbers are known to be even higher in Europe [23,24]. In our center, the use of these techniques is still low.

Atrial fibrillation was the most common arrhythmia, recorded in 22% during the study follow-up, similar to what the evidence states where AF is present in 25–53%, with an annual incidence of 4%/year contributing to a decreased quality of life and risk of systemic thromboembolism [22,25,26]. These patients do not tolerate losing the atrial kick in addition to the frequent diastolic dysfunction; therefore, aggressive rate control or restoration of sinus rhythm is crucial. In this setting, oral anticoagulation is mandatory (unless contraindicated) for patients with HCM irrespective of risk-scoring systems due to the higher risk of thromboembolism [27].

Cardiovascular mortality in these patients is most frequently due to HF, followed by SCD and stroke-related death. During follow-up, six patients (7.3%) died. These data are comparable to previous studies [11,12]. Mortality varies according to the genotype; patients with sarcomeric HCM are diagnosed earlier in life and have the worst prognosis [28]; also in addition, women tend to be diagnosed later in life, with more symptomatic heart failure and a higher mortality rate. Ventricular arrhythmias and SCD were presented in 10.7% and 2.4% of patients, respectively, the latter with reported annual rates of approximately 0.5–1% [29].

In the study by Fernandez et al. in which they evaluated patients with hypertrophic cardiomyopathy and left ventricular systolic impairment (ILVSF), it was found that during follow-up, 14 patients (58%) with ILVSF reached the combined end point (one patient [4.2%] died from heart failure and thirteen [54%] underwent heart transplant) compared to three patients (0.8%) with normal systolic function (*p* = 0.001) [8]. In our cohort, the rate of these outcomes is not high, probably because most of the patients had a preserved ejection fraction.

HCM has predominantly been considered an autosomal dominant genetic disease, although de novo mutations explain some cases and, less frequently, autosomal recessive heredity. The genetic study may play a role in stratifying the prognosis of HCM patients. Genetic testing was only performed on three patients. The 2018 Heart Failure Society of America guideline on cardiomyopathies recommends genetic counseling for all patients with cardiomyopathy and their family members and that genetic testing should be offered to all patients diagnosed with all recognized forms of cardiomyopathy [30]. Our study has patients evaluated since 2010. The genetic tests in our country at that time were limited, and currently, access to these tests is still difficult due to the cost.

HCM is caused by a variety of mutations in genes encoding contractile proteins of the cardiac sarcomere, especially in cardiac myosin heavy chain beta (MYH7), myosin binding protein C (MYBPC3), and cardiac troponin T (TNNT2). To date, over 700 individual mutations have been identified [31]. With respect to specific genes in our study, pathogenic TTR variants are rare in carefully assessed HCM patients and may occur in double heterozygosity with pathogenic sarcomere variants [32]. About 2–7% of familial cardiomyopathy cases are caused by a mutation in the gene encoding cardiac troponin I (TNNI3). A. van den Wijngaard et al. described in their study the majority of Dutch TNNI3 mutations were associated with a HCM phenotype [33]. One patient had TTN mutation. While TTN truncation mutations are common in DCM, there is evidence that TTN truncations are rare in the HCM phenotype, with a frequency similar to control populations [34]. Using high-throughout sequencing in 142 HCM probands, Lopes et al. found 219 TTN rare variants with 209 being novel missense variants [35]. However, this cohort of individuals potentially had a sarcomeric gene mutation that likely caused HCM, and the actual pathogenic role of these TTN variants is unknown.

Several limitations should be considered when interpreting our data, including the retrospective cohort design, which is more susceptible to the effects of confounding and bias, and the fact that it is a single-institution study. We think that dilated cardiomyopathy is more common than HCM because our hospital is a center for patients with advanced heart failure; this could account for the low frequency of HCM and add extra selection bias to the population. Due to the observational nature of this study, we cannot exclude the presence of infiltrative cardiomyopathies, such as amyloidosis, sarcoidosis, Fabry disease, and Dannon disease.

Future studies, providing complete clinical information in combination with family history, echocardiographic and CMR parameters, and genetic testing would better clarify and characterize the HCM phenotype. A register of HCM patients should be established through multicenter efforts to identify the unique characteristics of this illness in Latin America and contribute to the reduction of morbidity and mortality in this population.

## 5. Conclusions

HCM is a complex and heterogeneous disorder, presenting significant morbidity and mortality. This registry provides comprehensive data on the disease course and management in a developing country.

## Figures and Tables

**Table 1:** Baseline characteristics and treatment.

Variable	HCM (*n* = 82)
Age at clinical diagnosis, *n*	
Median (IQR)	49.0 (37.5–61.0)
Gender, *n*	
Male, *n* (%)	55 (67.1%)
Family history of cardiomyopathy, *n*, (%)	12 (25.0%)
Family history of cardiomyopathy by level of consanguinity, *n*	
First degree, *n* (%)	8 (66.7%)
Second degree, *n* (%)	3 (25.0%)
**NYHA functional class, *n* (%)**	
Class I	43 (59.7%)
Class II	17 (23.6%)
Class III	10 (13.9%)
Class IV	2 (2.8%)
Angina, *n* (%)	16 (19.5%)
Dyspnea, *n* (%)	31 (37.8%)
Palpitations, *n* (%)	9 (11.0%)
Syncope, *n* (%)	12 (14.6%)
**Personal history and comorbidities, *n* (%)**	
Atrial fibrillation	11 (15.1%)
Stroke	7 (9.5%)
Diabetes mellitus type 2	9 (11.2%)
Arterial hypertension	24 (30.4%)
Dyslipidemia	17 (21.2%)
Overweight/Obesity	6 (7.6%)
Chronic kidney disease	3 (3.8%)
Hypothyroidism	7 (8.8%)
Smoking	9 (17.0%)
**Pharmacologic treatment, *n* (%)**	
Beta-blockers	58 (85.3%)
Diuretics, oral	13 (19.1%)
ACE-inhibitors	1 (1.5%)
Angiotensin II receptor blockers	17 (25.0%)
Mineralocorticoid receptor antagonists	7 (10.3%)
Calcium channel blockers	19 (27.9%)
Acetylsalicylic acid	8 (11.8%)
SGLT2 inhibitors	1 (1.5%)
ARNI	2 (2.9%)
Statins	13 (19.1%)
Oral anticoagulants	14 (20.5%)
Vitamin K antagonists	4 (5.9%)
Apixaban	5 (7.4%)
Rivaroxaban	4 (5.9%)
Non-specified	1 (1.5%)
**Non-pharmacological treatment *n* (%)**	
Angiography	7 (19.4%)
Alcohol septal ablation	5 (6.1%)
Septal myectomy	4 (4.9%)
Ventricular assist device	1 (1.3%)
Cardiac resynchronization therapy	2 (2.4%)
Implantable cardioverter-defibrillators	29 (35.4%)

NYHA: New York Heart Association; ACE: angiotensin-converting enzyme); SGLT-2: sodium-glucose cotransporter-2; ARNI: angiotensin receptor/neprilysin inhibitor.

**Table 2:** Echocardiographic Characteristics.

Variable, *n* (%)	HCM (*n* = 82)
Ejection fraction *n* = 73	
Reduced (<40%)	4 (5.8%)
Slightly reduced (40–49%)	4 (5.8%)
Preserved (>50%)	65 (89%)
Global longitudinal strain, median (IQR)	−13.4 (−18.1, −11.7)
Wall diameters	
Abnormal interventricular septum (>9 mm female; >10 mm male)	63 (100%)
Abnormal posterior wall (>9 mm female; >10 mm male)	36 (76.6%)
HCM subtype according to LV hypertrophy pattern	
Asymmetric septal	37 (64.9%)
Concentric	16 (28.1%)
Predominantly apical	4 (7.0%)
No data	25/82 (30.5%)
Left atrial volume indexed	53 (65.4%)
Normal: 16 to 34 mL/m^2^	8 (15.1%)
Slightly abnormal: 35 to 41 mL/m^2^	20 (37.7%)
Moderately abnormal: 42 to 48 mL/m^2^	9 (17.0%)
Severely abnormal: >48 mL/m^2^	16 (30.2%)
E/A ratio	
Normal (0.8–2)	20 (69%)
Abnormal (>2)	9 (31.0%)
Right atrial area	
Normal ≤19 cm^2^	23(57.5%)
Abnormal >19 cm^2^	17(42.5%)
TAPSE	
Normal >17	32 (91.4%)
Decreased <17	3 (8.6%)
S’ wave	
Normal	18 (78.3%)
Abnormal	5 (21.7%)
Valvular heart disease	
Mitral regurgitation	43 (52.4%)
Aortic insufficiency	6 (7.3%)
Tricuspid insufficiency	34 (41.5%)
Aortic stenosis	4 (4.9%)
Mitral stenosis	0 (0.0%)
LV outflow tract gradient	
Normal	15 (27.3%)
<30 mmHg	11 (20.0%)
30–49 mmHg	8 (14.5%)
>50 mmHg	21 (38.2%)
Unreported	27/82 (32.9%)
SAM	21 (47.7%)
Pericardial effusion	2 (3.7%)

Asymmetric septal hypertrophy is considered when there is a pattern of septal thickness with the free LV wall >1.3/1.0. TAPSE: acronym for measurement of the tricuspid ring systolic excursion; LV: left ventricle; SAM: acronym for systolic anterior motion of the mitral valve.

## Data Availability

Data supporting this study are included within the article and/or supporting materials.

## References

[1] Elliott P.M., Anastasakis A., Borger M.A., Borggrefe M., Cecchi F., Charron P., Hagege A.A., Lafont A., Limongelli G., Mahrholdt H. (2014). 2014 ESC Guidelines on diagnosis and management of hypertrophic cardiomyopathy: The Task Force for the Diagnosis and Management of Hypertrophic Cardiomyopathy of the European Society of Cardiology (ESC). Eur. Heart J..

[2] Elliott P., Andersson B., Arbustini E., Bilinska Z., Cecchi F., Charron P., Dubourg O., Kühl U., Maisch B., McKenna W.J. (2008). Classification of the cardiomyopathies: A position statement from the european society of cardiology working group on myocardial and pericardial diseases. Eur. Heart J..

[3] Zipes D.P. (2018). Braunwald’s Heart Disease: A Textbook of Cardiovascular Medicine. BMH Med. J..

[4] McKenna W.J., Judge D.P. (2021). Epidemiology of the inherited cardiomyopathies. Nat. Rev. Cardiol..

[5] Marian A.J., Braunwald E. (2017). Hypertrophic Cardiomyopathy: Genetics, Pathogenesis, Clinical Manifestations, Diagnosis, and Therapy. Circ. Res..

[6] Bocchi E.A., Arias A., Verdejo H., Diez M., Gómez E., Castro P. (2013). The Reality of Heart Failure in Latin America. J. Am. Coll. Cardiol..

[7] Bocchi E.A. (2013). Heart Failure in South America. Curr. Cardiol. Rev..

[8] Fernández A., Vigliano C.A., Casabé J.H., Diez M., Favaloro L.E., Guevara E., Favaloro R.R., Laguens R.P. (2011). Comparison of Prevalence, Clinical Course, and Pathological Findings of Left Ventricular Systolic Impairment Versus Normal Systolic Function in Patients with Hypertrophic Cardiomyopathy. Am. J. Cardiol..

[9] Espinola-Zavaleta N., Vega A., Basto D.M., Alcantar-Fernández A.C., Lans V.G., Soto M.E. (2015). Survival and Clinical Behavior of Hypertrophic Cardiomyopathy in a Latin American Cohort in Contrast to Cohorts from the Developed World. J. Cardiovasc. Ultrasound.

[10] Maron B.J., Towbin J.A., Thiene G., Antzelevitch C., Corrado D., Arnett D., Moss A.J., Seidman C.E., Young J.B. (2006). Contemporary definitions and classification of the cardiomyopathies: An American Heart Association Scientific Statement from the Council on Clinical Cardiology, Heart Failure and Transplantation Committee; Quality of Care and Outcomes Research and Functional Genomics and Translational Biology Interdisciplinary Working Groups; and Council on Epidemiology and Prevention. Circulation.

[11] Ho C.Y., Day S.M., Ashley E.A., Michels M., Pereira A.C., Jacoby D., Cirino A.L., Fox J.C., Lakdawala N.K., Ware J. (2018). Genotype and Lifetime Burden of Disease in Hypertrophic Cardiomyopathy: Insights from the Sarcomeric Human Cardiomyopathy Registry (SHaRe). Circulation.

[12] Cardim N., Brito D., Lopes L.R., Freitas A., Araújo C., Belo A., Gonçalves L., Mimoso J., Olivotto I., Elliott P. (2018). The Portuguese Registry of Hypertrophic Cardiomyopathy: Overall results. Rev. Port. Cardiol..

[13] Neubauer S., Kolm P., Ho C.Y., Kwong R.Y., Desai M.Y., Dolman S.F., Appelbaum E., Desvigne-Nickens P., DiMarco J.P., Friedrich M.G. (2019). Distinct Subgroups in Hypertrophic Cardiomyopathy in the NHLBI HCM Registry. J. Am. Coll. Cardiol..

[14] Maron B.J., Desai M.Y., Nishimura R.A., Spirito P., Rakowski H., Towbin J.A., Rowin E.J., Maron M.S., Sherrid M.V. (2022). Diagnosis and Evaluation of Hypertrophic Cardiomyopathy: JACC State-of-the-Art Review. J. Am. Coll. Cardiol..

[15] Lang R.M., Badano L.P., Mor-Avi V., Afilalo J., Armstrong A., Ernande L., Flachskampf F.A., Foster E., Goldstein S.A., Kuznetsova T. (2015). Recommendations for Cardiac Chamber Quantification by Echocardiography in Adults: An Update from the American Society of Echocardiography and the European Association of Cardiovascular Imaging. Eur. Heart J. Cardiovasc. Imaging.

[16] Wigle E.D., Rakowski H., Kimball B.P., Williams W.G. (1995). Hypertrophic Cardiomyopathy: Clinical spectrum and treatment. Circulation.

[17] Jan M.F., Todaro M.C., Oreto L., Tajik A.J. (2016). Apical hypertrophic cardiomyopathy: Present status. Int. J. Cardiol..

[18] Kitaoka H., Doi Y., A Casey S., Hitomi N., Furuno T., Maron B.J. (2003). Comparison of prevalence of apical hypertrophic cardiomyopathy in Japan and the United States. Am. J. Cardiol..

[19] Huang G., Fadl S.A., Sukhotski S., Matesan M. (2020). Apical variant hypertrophic cardiomyopathy “multimodality imaging evaluation”. Int. J. Cardiovasc. Imaging.

[20] Maron M.S., Olivotto I., Zenovich A.G., Link M.S., Pandian N.G., Kuvin J.T., Nistri S., Cecchi F., Udelson J.E., Maron B.J. (2006). Hypertrophic Cardiomyopathy Is Predominantly a Disease of Left Ventricular Outflow Tract Obstruction. Circulation.

[21] Unger T., Borghi C., Charchar F., Khan N.A., Poulter N.R., Prabhakaran D., Ramirez A., Schlaich M., Stergiou G.S., Tomaszewski M. (2020). 2020 International Society of Hypertension Global Hypertension Practice Guidelines. Hypertension.

[22] Lee P.T., Dweck M.R., Prasher S., Shah A., Humphries S.E., Pennell D.J., Montgomery H.E., Payne J.R. (2013). Left Ventricular Wall Thickness and the Presence of Asymmetric Hypertrophy in Healthy Young Army Recruits: Data from the LARGE heart study. Circ. Cardiovasc. Imaging.

[23] Ommen S.R., Mital S., Burke M.A., Day S.M., Deswal A., Elliott P., Evanovich L.L., Hung J., Joglar J.A., Kantor P. (2020). 2020 AHA/ACC Guideline for the Diagnosis and Treatment of Patients with Hypertrophic Cardiomyopathy. J. Am. Coll. Cardiol..

[24] Maron B.J., Yacoub M., Dearani J.A. (2011). Benefits of surgery in obstructive hypertrophic cardiomyopathy: Bring septal myectomy back for European patients. Eur. Heart J..

[25] Kim L.K., Swaminathan R.V., Looser P., Minutello R.M., Wong S.C., Bergman G., Naidu S.S., Gade C.L.F., Charitakis K., Singh H.S. (2016). Hospital Volume Outcomes After Septal Myectomy and Alcohol Septal Ablation for Treatment of Obstructive Hypertrophic Cardiomyopathy. JAMA Cardiol..

[26] van Velzen H.G., Theuns D.A., Yap S.-C., Michels M., Schinkel A.F. (2017). Incidence of Device-Detected Atrial Fibrillation and Long-Term Outcomes in Patients With Hypertrophic Cardiomyopathy: US nationwide inpatient database, 2003–2011. Am. J. Cardiol..

[27] Wilke I., Witzel K., Münch J., Pecha S., Blankenberg S., Reichenspurner H., Willems S., Patten M., Aydin A. (2016). High Incidence of De Novo and Subclinical Atrial Fibrillation in Patients with Hypertrophic Cardiomyopathy and Cardiac Rhythm Management Device. J. Cardiovasc. Electrophysiol..

[28] Guttmann O.P., Rahman M.S., O’Mahony C., Anastasakis A., Elliott P.M. (2014). Atrial fibrillation and thromboembolism in patients with hypertrophic cardiomyopathy: Systematic review. Heart.

[29] Moore B., Semsarian C., Chan K.H., Sy R.W. (2019). Sudden Cardiac Death and Ventricular Arrhythmias in Hypertrophic Cardiomyopathy. Heart Lung Circ..

[30] Hershberger R.E., Givertz M.M., Ho C.Y., Judge D.P., Kantor P.F., McBride K.L., Morales A., Taylor M.R., Vatta M., Ware S.M. (2018). Genetic Evaluation of Cardiomyopathy—A Heart Failure Society of America Practice Guideline. J. Card. Fail..

[31] Colombo M.G., Botto N., Vittorini S., Paradossi U., Andreassi M.G. (2008). Clinical utility of genetic tests for inherited hypertrophic and dilated cardiomyopathies. Cardiovasc. Ultrasound.

[32] Lopes L.R., Futema M., Akhtar M.M., Lorenzini M., Pittman A., Syrris P., Elliott P.M. (2019). Prevalence of *TTR* variants detected by whole-exome sequencing in hypertrophic cardiomyopathy. Amyloid.

[33] Wijngaard A.v.D., Volders P., Van Tintelen J.P., Jongbloed J.D.H., Berg M.P.v.D., Deprez R.H.L., Mannens M.M.A.M., Hofmann N., Slegtenhorst M., Dooijes D. (2011). Recurrent and founder mutations in the Netherlands: Cardiac Troponin I (TNNI3) gene mutations as a cause of severe forms of hypertrophic and restrictive cardiomyopathy. Neth. Heart J..

[34] Gigli M., Begay R.L., Morea G., Graw S.L., Sinagra G., Taylor M.R.G., Granzier H., Mestroni L. (2016). A Review of the Giant Protein Titin in Clinical Molecular Diagnostics of Cardiomyopathies. Front. Cardiovasc. Med..

[35] Lopes L.R., Zekavati A., Syrris P., Hubank M., Giambartolomei C., Dalageorgou C., Jenkins S., McKenna W., Plagnol V., Elliott P.M. (2013). Genetic complexity in hypertrophic cardiomyopathy revealed by high-throughput sequencing. J. Med. Genet..

